# Proteomic Profiles and Biological Processes of Relapsed vs. Non-Relapsed Pediatric Hodgkin Lymphoma

**DOI:** 10.3390/ijms21062185

**Published:** 2020-03-22

**Authors:** Ombretta Repetto, Valli De Re, Lara Mussolin, Massimo Tedeschi, Caterina Elia, Maurizio Bianchi, Salvatore Buffardi, Alessandra Sala, Roberta Burnelli, Maurizio Mascarin

**Affiliations:** 1 Facility of Bio-Proteomics, Immunopathology and Cancer Biomarkers, Centro di Riferimento Oncologico (CRO) di Aviano, IRCCS, 33081 Aviano (PN), Italy; massimo.tedeschi@cro.it; 2 Clinic of Pediatric Haemato-Oncology, Department of Women’s and Children’s Health, Institute of Paediatric Research—Fondazione Città della Speranza, University of Padua, 35122 Padua, Italy; lara.mussolin@unipd.it; 3 Pediatric Radiotherapy Unit, Centro di Riferimento Oncologico (CRO) di Aviano, IRCCS, 33081 Aviano (PN), Italy; eliacaterina@libero.it (C.E.); mascarin@cro.it (M.M.); 4 Pediatric Onco-Hematology and Stem Cell Transplant Division, City of Health and Science, Regina Margherita Children’s Hospital, 10126 Turin, Italy; maurizio.bianchi@unito.it; 5 Paediatric Haemato-Oncology Department, Santobono-Pausilipon Children’s Hospital, 80129 Naples, Italy; salvatorebuffardi@hotmail.it; 6 Department of Paediatrics, Ospedale San Gerardo, University of Milano-Bicocca, Fondazione MBBM, 20052 Monza, Italy; ale.sala@asst-monza.it; 7 Pediatric Oncology University Hospital, Sant’Anna Hospital, 44124 Ferrara, Italy; r.burnelli@ospfe.it

**Keywords:** biomarker, cancer, label-free quantification, pediatric Hodgkin lymphoma, plasma, protein mass spectrometry, proteomics, relapse

## Abstract

The identification of circulating proteins associated with relapse in pediatric Hodgkin lymphoma (HL) may help develop predictive biomarkers. We previously identified a set of predictive biomarkers by difference gel electrophoresis. Here we used label-free quantitative liquid chromatography-mass spectrometry (LC-MS/MS) on plasma collected at diagnosis from 12 children (age 12–16 years) with nodular sclerosis HL, including six in whom the disease relapsed within 5 years of treatment in the LH2004 trial. Plasma proteins were pooled in groups of three, separately for non-relapsing and relapsing HL, and differentially abundant proteins between the two disease states were identified by LC-MS/MS in an explorative and validation design. Proteins with a fold change in abundance >1.2 or ≤0.8 were considered “differentially abundant”. LC-MS/MS identified 60 and 32 proteins that were more abundant in non-relapsing and relapsing HL plasma, respectively, in the explorative phase; these numbers were 39 and 34 in the validation phase. In both analyses, 11 proteins were more abundant in non-relapsing HL (e.g., angiotensinogen, serum paraoxonase/arylesterase 1, transthyretin), including two previously identified by difference gel electrophoresis (antithrombin III and α-1-antitrypsin); seven proteins were more abundant in relapsing HL (e.g., fibronectin and thrombospondin-1), including two previously identified proteins (fibrinogen β and γ chains). The differentially abundant proteins participated in numerous biological processes, which were manually grouped into 10 biological classes and 11 biological regulatory subclasses. The biological class Lipid metabolism, and its regulatory subclass, included angiotensinogen and serum paraoxonase/arylesterase 1 (more abundant in non-relapsing HL). The biological classes Immune system and Cell and extracellular matrix architecture included fibronectin and thrombospondin-1 (more abundant in relapsing HL). These findings deepen our understanding of the molecular scenario underlying responses to therapy and provide new evidence about these proteins as possible biomarkers of relapse in pediatric HL.

## 1. Introduction

Pediatric Hodgkin lymphoma (HL) represents 6% of all childhood cancers and has a 5-year survival rate of approximately 90–95% [1]. The incidence of relapsed pediatric HL is around 10% for patients with early stage disease [2] and 15–20% for those with advanced stage disease [3]. There is currently great interest in finding predictive biomarkers to help identify subgroups of patients at risk of treatment failure or relapse. Several studies use a candidate-protein approach with described putative biomarkers measurable by enzyme-linked immunosorbent assay in pretreatment blood or serum and are predictive of treatment outcome (reviewed in [4]). These proteins include CD54 [5], heparanase [6], and VEGF [7].

Another approach to finding biomarkers, not limited to already known candidates, is provided by proteomics. For example, Qi et al. 2008 [8] used mass spectrometry (MS)-based proteomic profiling with surface enhanced laser desorption/ionization to identify serum proteins discriminating advanced HL stages, and α-1-antitrypsin emerged as a candidate biomarker for high grade (III/IV) pediatric HL. Kamper et al. 2011 [9] analyzed tissue samples with two-dimensional electrophoresis (2DE) combined with liquid chromatography-tandem MS (LC-MS/MS), and found an association between high levels of the immune-suppressive protein galectin-1 and adverse outcome in patients ≤61 years. We used difference gel electrophoresis (DIGE), a modification of 2DE, to identify plasma proteins specifically associated with either disease relapse after treatment (fibrinogen α and β chains, complement C3, and ceruloplasmin) or no relapse (α-1-antitrypsin, apolipoprotein A-IV; antithrombin II, inter-α trypsin inhibitor, and vitronectin) in pediatric HL [10]. In DIGE, proteins are covalently tagged with fluorescent dyes, such as the cyanine dyes cy2, cy3 and cy5, which form an amide bond with the ɛ-amino group of lysine residues on proteins [11]. DIGE is a powerful approach for comparing and quantifying proteins. However, when applied to the highly variable plasma matrix, DIGE has difficulty separating extremely acidic, basic, or hydrophobic proteins, and rare or comigrating proteins may not be detected [12].

The problems of DIGE in analyzing complex plasma samples can be overcome by LC-MS. LC-MS enables the detection of thousands of molecules in a fast, sensitive, and high-throughput way. Different methods for relative quantification are available. In label-free methods, native proteins are detected by spectral counting or precursor-based quantification [13]. In label-based methods, proteins are labeled either metabolically (e.g., adding a label to cells in culture) or chemically (e.g., proteins are chemically or enzymatically tagged in vitro) before LC-MS [14]. Label-free MS protocols are advantageous because of their capacity to detect rare peptides in samples with a high dynamic range of quantification, especially when substantial changes in protein content are investigated [13,15,16]. Label-free quantitative LC-MS/MS has proven useful in the discovery of markers for ovarian [17], breast [18], gastric [19] and colorectal [20] cancers, but not yet for pediatric HL.

In this quantitative proteomic study, we used label-free LC-MS/MS to profile plasma proteins from pediatric HL patients and identify those that are differentially abundant between patients who relapsed within 5 years of treatment and those who did not. The study also attempted to validate, with a different method, the biomarkers that we previously reported to be associated with HL relapse [10]. Finally, the study aimed to identify biological processes associated with relapse in pediatric HL based on these differentially abundant plasma proteins.

## 2. Results

### 2.1. Plasma Protein Profiling

Label-free quantitative LC-MS/MS was performed on plasma proteins from six children with relapsing HL and six with non-relapsing HL (Table 1). All patients had nodular sclerosis HL. Nine patients had stage 2 disease and three had stage 4 disease, while four of the 12 had at least one systemic symptom. For the purposes of this study, the patients were divided into equal groups for explorative and validation phases.

In the explorative analysis, LC-MS/MS identified 60 proteins as being more abundant in non-relapsing HL and 32 proteins more abundant in relapsing HL (Table 2). In the validation analysis, 39 and 34 proteins were identified as being more abundant in non-relapsing HL and relapsing HL, respectively (Table S1). The validation analysis confirmed the differential abundance for 11 and 7 proteins, respectively (total, 18 proteins). In non-relapsing HL, the more abundant proteins (and their gene symbols) were (in gene alphabetical order): angiotensinogen (*AGT*), complement C1q subcomponent subunit B (*C1QB*), EGF-containing fibulin-like extracellular matrix protein 1 (*EFEMP1*), fibulin-1 (*FBLN1*), histidine-rich glycoprotein (*HRG*), serum paraoxonase/arylesterase 1 (*PON1*), pregnancy zone protein (*PZP*), α-1-antitrypsin (*SERPINA1*), antithrombin III (*SERPINC1*), α-2-antiplasmin (*SERPINF2*), and transthyretin (*TTR*). In relapsing HL, they were: α-1B-glycoprotein (*A1BG*), complement C1s subcomponent (*C1S*), fibrinogen β chain (*FGB*), fibrinogen γ chain (*FGG*), fibronectin (*FN1*), thrombospondin-1 (*THBS1*), and talin-1 (*TLN1*).

Among the validated differentially abundant proteins, four had previously been identified by us (using DIGE) as being differentially abundant in non-relapsing vs. relapsing HL [10]. Two of them had similarly been found to be more abundant in non-relapsing HL (antithrombin III and α-1-antitrypsin), while two were similarly more abundant in relapsing HL (fibrinogen β and γ chains).

**Figure 1:**
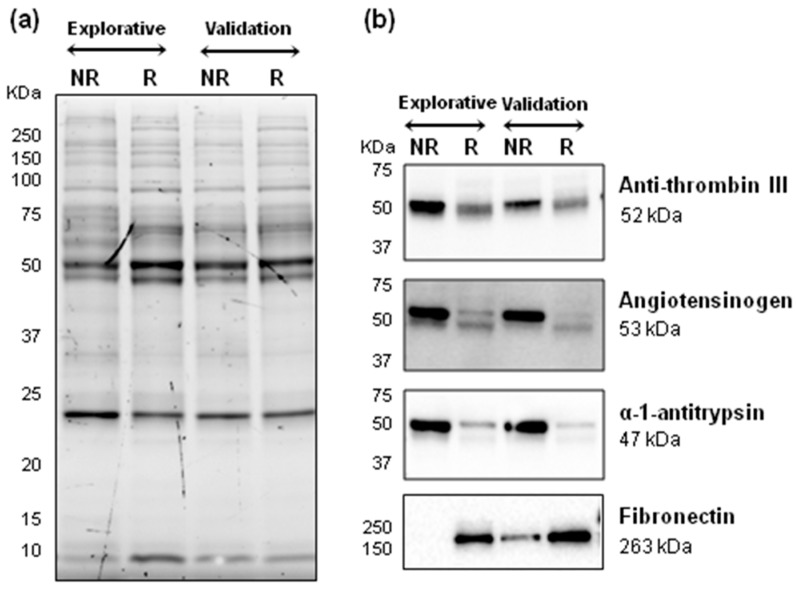
Immunoblotting validation of differential abundance of plasma proteins between non-relapsing (NR) and relapsing (R) HL (pools of three samples each). (**a**) Chemidoc image of gel before transfer of proteins to nitrocellulose membranes. (**b**) Blots probed with primary antibodies against four differentially abundant proteins.

The differential abundance of the proteins identified here was examined by immunoblotting of pooled plasma proteins for four selected proteins. This analysis confirmed the higher levels of antithrombin III, angiotensinogen and α-1-antitrypsin in non-relapsing HL plasma, and of fibronectin in relapsing HL plasma (Figure 1).

### 2.2. Functional Annotation of Differentially Abundant Proteins

**Figure 2:**
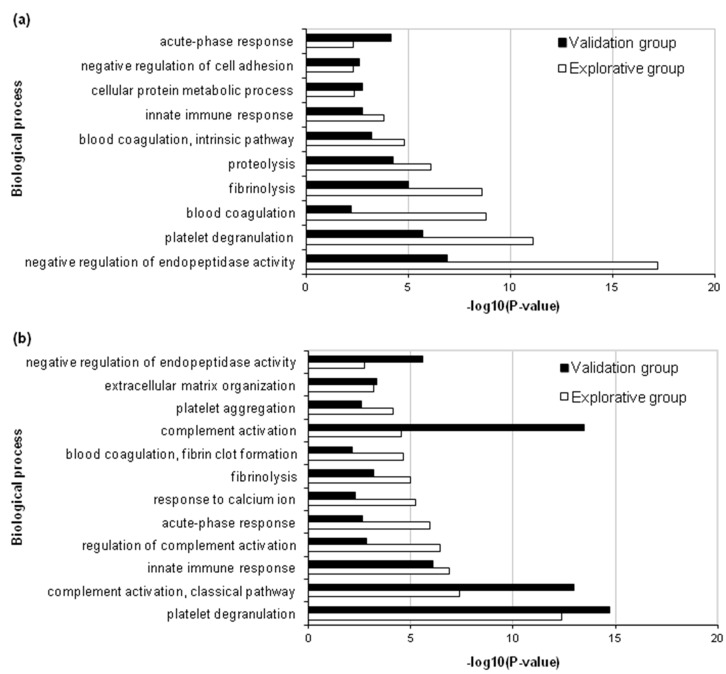
Most significant (*p* < 0.01) biological processes associated with differentially abundant plasma proteins according to DAVID Bioinformatics Resources and common to the explorative and validation analyses. (**a**) Non-relapsing HL. (**b**) Relapsing HL.

DAVID Bioinformatics Resources were used to identify gene ontology (GO) biological processes related to the differently abundant proteins of the explorative and validation analyses (Table S2). For non-relapsing HL, the most significant process in the explorative analysis was “negative regulation of endopeptidase activity” (GO:0010951), followed by “platelet degranulation” (GO:0002576), “blood coagulation” (GO:0007596) and “fibrinolysis” (GO:0042730) (Figure 2a). For relapsing HL, the most significant process was “platelet degranulation”, followed by “complement activation, classical pathway” (GO:0006958), “innate immune response” (GO:0045087) and “regulation of complement activation” (GO:0030449) (Figure 2b). Similar results were obtained in the validation analysis.

The functional enrichments of all proteins identified in the explorative and validation analyses were then examined with STRING, focusing on biological processes. Total counts of gene sets enriched with differentially abundant proteins revealed 282 and 226 GO biological processes affected in non-relapsing and relapsing HL, respectively, in the explorative analysis, and 186 and 156 GO biological processes affected in non-relapsing and relapsing HL, respectively, in the validation one. Most proteins were associated with more than one gene set (STRING_EnrichmentProcess_Explorative&ValidationAnalyses_RawData). With respect to DAVID, STRING analysis revealed more biological processes.

### 2.3. Analysis of Biological Processes

To characterize the differential proteins in terms of their biological processes and investigate the biological hallmarks of relapse, we did an ad hoc analysis of GO biological processes. To this aim, we sorted all the biological processes implicated in both non-relapsing (*n* = 468) and relapsing (*n* = 381) HL into groups with similar GO term descriptions found with STRING (PPI enrichment *p*-value <1.0e-16 and FDR < 0.05), forming 10 biological classes: Immune system, Regulation, Transport and homeostasis, Coagulation, Fibrinolysis, Vascularization, Response, Cell and ECM organization, Lipid metabolism, and Protein metabolism (Table S3). The classes included between 2 (Fibrinolysis class) and 167 biological processes (Regulation class). Because the Regulation class had many more processes than the other classes, it was subdivided into 11 biological regulatory subclasses: Immune system, Transport and homeostasis, Coagulation, Fibrinolysis, Vascularization, Response, Cell and ECM organization, Cell death, Lipid metabolism, Protein metabolism and Signaling; these subclasses included between 4 (Fibrinolysis subclass) and 38 (Protein metabolism subclass) biological processes.

All differentially abundant proteins in relapsing and non-relapsing HL were associated with these new biological classes and subclasses through the corresponding GO biological processes (BiologicalClasses&Subclasses_RawData). This new classification of processes is compared to that provided by DAVID, for the 18 validated differentially abundant proteins, in Table 3. The two classifications agree overall and provide complementary information: while DAVID assigns, to each protein, the individual biological processes, our approach assigns grouped biological processes. Moreover, our approach associates the proteins with other biological processes not considered by DAVID, an analysis that was more restrictive (*p* < 0.01).

**Figure 3:**
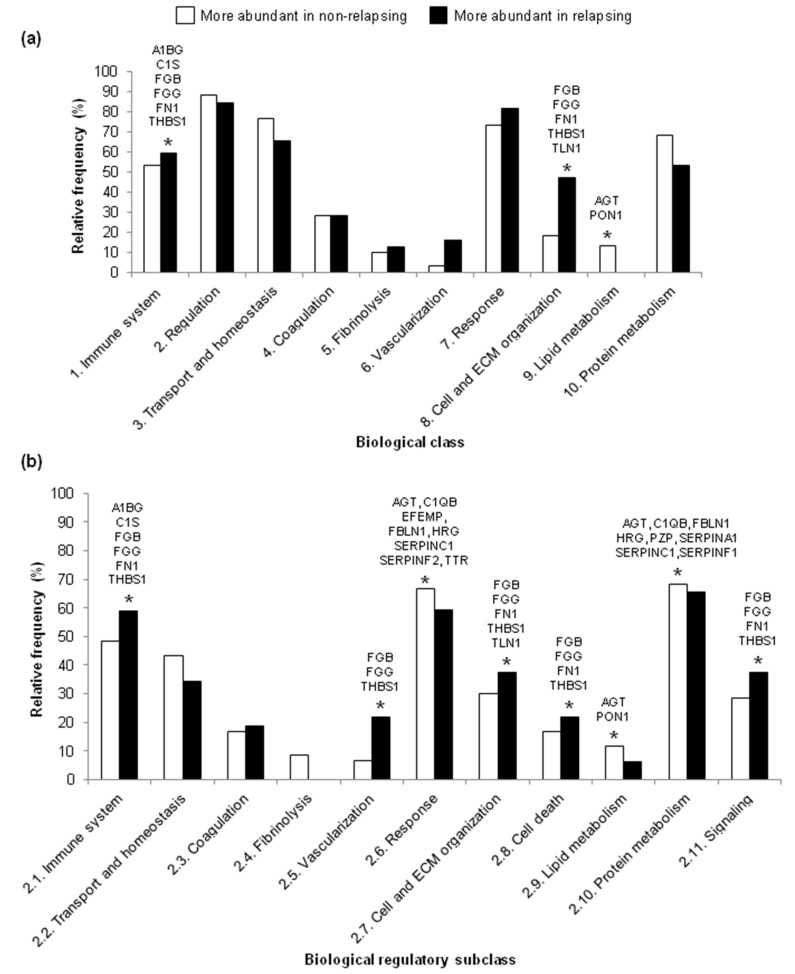
Relative frequencies of the differentially abundant proteins in 10 biological classes (**a**) and 11 biological regulatory subclasses (**b**) in the explorative analysis of pediatric HL patients. Gene symbols above bars marked with an asterisk refer to proteins involved in those processes that were also identified in the validation analysis. Corresponding data for the validation analysis are shown in Figure S1.

Concerning the differentially abundant proteins in our biological classes, the relative frequencies of proteins were higher in the non-relapsing HL group than the relapsing HL group for the Regulation and Lipid metabolism biological classes in both the explorative (Figure 3a) and validation (Figure S1a) analyses. In contrast, the relative frequencies were higher in the relapsing HL group than the non-relapsing HL group for the Immune system and Cell and ECM organization biological classes in both analyses (Figure 3a and Figure S1a). Some of the proteins in these biological classes of interest were identified in both groups of patients as being more abundant in either non-relapsing HL (*AGT* and *PON1*) or relapsing HL (*A1BG, C1S, FN1, FGB, FGG* and *THBS1*) (Figure 3a and Figure S1a).

In both the explorative and validation analyses, proteins included in the Response, Protein metabolism and Lipid metabolism biological regulatory subclasses were more abundant in the non-relapsing HL group than in the relapsing HL group (Figure 3b and Figure S1b). In contrast, proteins in the Immune system, Vascularization, Cell and ECM organization, Cell death and Signaling biological regulatory subclasses were more abundant in the relapsing HL group than non-relapsing HL group (Figure 3b and Figure S1b). Some of the proteins in these biological regulatory subclasses of interest were identified in both groups of patients as being more abundant in either non-relapsing HL (*AGT, C1QB, EFEMP, TTR, FBLN1, HRG, PZP, SERPINA1, SERPINC1, SERPINF2* and *PON1*) or relapsing HL (*A1BG, C1S, FGB, FGG, FN1* and *THBS1*).

## 3. Discussion

This LC-MS/MS proteomics study identified 18 plasma proteins whose levels at diagnosis of pediatric HL associated with relapse in both the explorative and validation phases. Of these proteins, 11 were more abundant in non-relapsing HL and 7 were more abundant in relapsing HL. Four of these proteins had been identified as being differentially abundant in our previous study [10]: here, we validated α-1-antitrypsin and antithrombin III as being more abundant in non-relapsing HL, and fibrinogen β and γ chains as being more abundant in relapsing HL. By immunoblotting, we confirmed the higher levels of α-1-antitrypsin, antithrombin III and angiotensinogen in non-relapsing HL, and fibronectin in relapsing HL. These confirmatory findings provide further evidence in support of these proteins as potential markers of the therapeutic response in pediatric HL.

Overall, the number of differentially abundant proteins found here by label-free quantitative LC-MS/MS is higher than what we found in our previous study using DIGE [10]. This difference may be methodological. With DIGE-MS, sample proteins are first labeled and then separated on a gel according to their isoelectric point and mass; proteins in spots that differ between different samples are identified after trypsin digestion and separation by MS [22]. This method, however, is not able to separate all proteins. With LC-MS/MS, sample proteins are first digested into peptides, which are then separated by LC and identified from their mass/charge ratio on MS [23]. The LC-based method, used here, is able to identify more proteins than the gel-based method, and therefore is better suited for the search for biomarkers.

DAVID interrogation of GO biological processes involving the differentially abundant proteins showed enrichment in the negative regulation of endopeptidase activities in non-relapsing HL. The differentially abundant proteins in this biological process (α-1-antitrypsin, antithrombin III, angiotensinogen, histidine-rich glycoprotein) are also involved in Blood coagulation and Fibrinolysis (α-1-antitrypsin, antithrombin III and histidine-rich glycoprotein), Platelet degranulation or activation (α-1-antitrypsin) and Cell adhesion (histidine-rich glycoprotein). During cancer onset and progression, the tumor microenvironment and the metabolic adaptation/reprogramming of tumor cells influence each other, leading to the dysregulation of various enzymes and to the activation of certain pathways by receptor-ligand interactions [24,25].

This study found that relapse-predicting proteins were enriched in the processes of Complement activation and Innate immune response. Together with the fibrinogen β and γ chains, which we already reported to be associated with relapse [10], complement C1s subcomponent also participates in these biological processes. In urothelial carcinoma, the high expression of C1S correlated with adverse clinicopathological parameters [26].

To get more insight into the biology of relapse in pediatric HL, we characterized all the differentially abundant proteins in terms of their biological processes. Our ad hoc grouping of biological processes found with STRING into 10 biological classes and 11 regulatory subclasses allowed us to observe that the differentially abundant proteins varied in relative number in a few biological classes and regulatory subclasses. The classes Regulation and Lipid metabolism and the regulatory subclass of Lipid metabolism associated with non-relapsing HL, while Immune system and Cell and ECM organization associated with relapsing HL. Moreover, a higher relative frequency of proteins implicated in regulation of Immune system, Vascularization, Cell and ECM organization, Cell death and Signaling was found in plasma of relapsing HL. In contrast, plasma of non-relapsing HL had more proteins involved in the regulation of Response, Lipid metabolism, and Protein metabolism. These data agree with those obtained with DAVID: in non-relapsing HL, the increase in Negative regulation of endopeptidase activity corresponds to the increase in the Regulation class and Protein metabolism subclass; in relapsing HL, the increase in Complement activation, Classical pathway and Innate immune response corresponds to Immune system class and subclass. Our classification revealed an increase in proteins associated with Lipid metabolism and Cell and ECM organization in non-relapsing and relapsing HL, respectively.

In general, leukemia and lymphoma patients have altered lipid metabolism, and in particular, lymphomagenesis is characterized by a decrease in circulating high-density lipoprotein (HDL) cholesterol [27,28]. This altered cholesterol metabolism reflects inflammation and abnormal endothelial vasoprotection [29]. A cross-sectional study that compared serum lipid profiles between children with HL and healthy children found an inverse association between HL and levels of both HDL and LDL cholesterol [30]. Therefore, data about lipid profiles (e.g., triglycerides, total cholesterol, LDL cholesterol) may help decipher the nature of proteins involved in the Lipid metabolism class found to be decreased in relapsing HL in this study. In particular, in plasma of relapsing HL, we found greater levels of angiotensinogen, which is involved in plasma lipoprotein particle remodeling and lipid metabolism [31,32], and in serum paraoxonase/arylesterase 1, recently described as an anti-inflammatory, antioxidant regulator of lipid metabolism [33].

Tumor progression depends on the ability of cancer cells to communicate with the ECM and to influence its biochemical and biomechanical properties [34]. Properties of the tumor microenvironment, including ECM and stromal compositions, influence the phenotype of tumor cells, and may alter vascular permeability, drug delivery and response to chemotherapy [35]. We found that some proteins in the biological class Cell and ECM organization were more abundant in non-relapsing than relapsing HL. In particular, the ECM-associated proteins fibronectin, fibrinogen, and thrombospondin-1 were more abundant in relapsing HL. Other ECM-associated proteins were more abundant in non-relapsing HL (e.g., fibulin-1, EGF-containing fibulin-like extracellular matrix protein 1). Fibulin-1, which is expressed mainly by cancer cells and only by some fibroblasts [36], has been reported to be overexpressed in lymph node biopsies from HL patients [37].

Cancer cells subvert the immune components of the tumor microenvironment in order to grow and spread, by activating certain signaling pathways. We found, in relapsing HL, a greater abundance of proteins involved in biological processes grouped in the Immune system class. Importantly, we observed a higher content of proteins involved in the Regulation of immune system (e.g., regulation of cytokine or interleukin-8 production, regulation of immune system/inflammatory process, regulation of macrophage activation) and Regulation of signaling (e.g., positive regulation of MAPK, ERK1/ERK2 cascades, tumor necrosis factor and toll-like receptor production). Indeed, HL is characterized by a dense inflammatory microenvironment [38].

HL cells use multiple mechanisms for immune escape and self-destruction avoidance [39,40,41]. In particular, in adult classic HL, immune escape is mediated by overexpression of programmed cell death 1 ligand 1 (PD-L1) on the surface of Hodgkin-Reed-Sternberg cells (reviewed in [39]). Overexpression in turn antagonizes the activity of PD-1-positive T cells and leads to tumor evasion of destruction by the immune system [42].

## 4. Materials and Methods

### 4.1. Research Ethics Statement

Plasma used in this study was obtained from the multicenter LH2004 clinical trial organized by A.I.E.O.P. (Associazione Italiana di Emato-Oncologia Pediatrica) for the treatment of pediatric HL and conducted in Italy from 1 June 2004 to 1 April 2014. The LH2004 trial was approved by both the Ethics Committee of CRO National Cancer Institute, Aviano (Italy) (prot. N. 206/D) and the Ethics Committee of the promoter center Azienda Ospedaliera Policlinico S. Orsola Malpighi, Bologna (Italy) (Prot N. 1103/2004), including the written informed consent for research on biological samples. The LH2004 trial was also approved by the HL Study Group of A.I.E.O.P. and by the Ethics Committee of each participating institution. The parents or legal guardians had given written informed consent for the plasma samples to be used for future research by researchers of the working groups involved in the trial.

### 4.2. Patients and Plasma Samples

This study used plasma samples from 12 pediatric HL patients enrolled in LH2004 [43]. These patients were selected for having had either a favorable (e.g., non-relapsing, *n* = 6) or unfavorable (relapsing, *n* = 6) response to treatment over a five-year period in the trial. Additional inclusion criteria were sex (equal numbers of boys and girls), age (between 12 and 16 years), nodular sclerosis histology (according to [44]) and stage (preferentially 2). Clinical data collected included stage according to [21], absence or presence of systemic symptoms (i.e., unexplained night sweating or weight loss of more than 10% over 6 months, or fever with temperature above 38 °C) [21] during the five-year follow-up, and LH2004 therapeutic group. Relapse was defined as the pathologically confirmed recurrence of HL.

Blood samples had been collected in sodium citrate vials at HL diagnosis, and plasma had been obtained and aliquoted at −80 °C.

Patients were divided into four groups of three each: (i) non-relapsing HL, explorative group; (ii) relapsing HL, explorative group; (iii) non-relapsing HL, validation group; and (iv) relapsing HL, validation group. The first two groups were used for the exploratory phase of our study, while the last two groups were used for validation of markers identified in the explorative phase.

### 4.3. Protein Extraction and Digestion

Protein was extracted from 200 μL plasma. First, we used the ProteoMiner Kit (Bio-Rad Laboratories, Hercules, CA, USA) to concentrate low-abundance proteins. Then, eluates from the kit were cleared of interfering solutes using the 2-D Clean-Up Kit (GE Healthcare, Uppsala, Sweden). The precipitate was resuspended in rehydration buffer (7 M urea, 2 M thiourea, 4% CHAPS, 0.5% *v/v* Pharmalytes ampholyte-containing buffer). Reagents were from GE Healthcare. Protein concentration was measured with the Bradford assay (Bio-Rad) and 2D Quant Kit (GE Healthcare).

Protein extracts were pooled within each group of three and digested in S-Trap spin columns (Protifi, Farmingdale NY, USA) accordingly to the manufacturer’s procedure. Briefly, 300 μg extract was mixed with 3% sodium dodecyl sulfate and 20 mM dithiothreitol, boiled, cooled to room temperature, and then alkylated with 40 mM iodoacetamide in the dark for 30 min. To each sample, phosphoric acid was added to a final concentration of 1.2% *v/v* together with six volumes of binding buffer (100 mM ammonium bicarbonate in 90% methanol). After gentle mixing, the solutions were loaded onto S-Traps and spun at 2000 rpm; the flow-through was collected and reloaded onto the S-Trap. This step was repeated three times more. Then each S-Trap was washed with the binding buffer three times. Finally, digestion buffer (50 mM ammonium bicarbonate) containing trypsin (30 µg per sample) was added to digest the proteins for 1 h at 47 °C. Hydrophilic peptides were eluted with 50 mM ammonium bicarbonate, 0.2% (*v/v*) aqueous formic acid while hydrophobic peptides were eluted with 50% acetonitrile, 0.2% (*v/v*) formic acid. These peptide solutions were combined for each pool of samples, lyophilized, and resuspended in 300 µL 0.2% formic acid. Reagents were from Sigma-Aldrich (St-Louis, MO, USA).

### 4.4. LC-MS/MS and Label-Free Proteomic Profiling

The peptide mixtures were analyzed by liquid chromatography-mass spectrometry (LC-MS/MS) by the Facility of Proteomics of CEINGE-Biotecnologie Avanzate (Naples, Italy) using the LTQ Orbitrap XL mass spectrometer equipped with ETD nano LC-MS/MS LIT-FITR (Thermo Fisher Scientific, Massachusetts, USA). Samples were analyzed in duplicate (technical replicates). They were loaded, concentrated, and desalted on a C18 Easy-Column (L = 2 cm, ID = 100 μm; cat. no. 03-052-619, Thermo Scientific SC001). They were then fractioned on a C18 reverse-phase capillary column (L = 20 cm, ID = 7.5 μm; cat. no. NS-AC-12, NanoSeparations, Niewkoop, Netherlands) at a flow rate of 250 nl/min in a gradient from 5% to 95% buffer B (eluent B: 0.2% formic acid in 95% acetonitrile; eluent A: 0.2% formic acid and 2% acetonitrile in ultrapure water) over 285 min. LC-MS/MS raw data were analyzed using MaxQuant software (www.maxquant.org). The MaxQuant peptide search engine Andromeda was used to identify proteins by matching peaks in the spectra to the theoretical fragment masses, and to perform label-free quantification by spectral counts (SpCs), which represent the abundance of each protein. Spectral counts of technical duplicates were averaged.

Fold change in protein abundance was calculated as *Rsc* [45], the log_2_ ratio of spectral counts between relapsing HL and non-relapsing HL groups. Differentially abundant proteins were defined as those with a fold change, calculated for at least three peptides, ≤0.8 or >1.2 in both the explorative and validation groups.

### 4.5. Immunoblotting

The levels of proteins found to be differentially abundant in both the explorative and validation groups were examined by immunoblotting of two pools of plasma proteins from both non-relapsing and relapsing patients (3 samples per pool). Protein (10 µg per sample) was fractionated on 12% Criterion TGX Stain-Free gels (Bio-Rad) and, after gel image acquisition with the Chemidoc system (Bio-Rad), the proteins were electrotransferred onto nitrocellulose membranes. The following primary antibodies were used: anti-antithrombin III [EP5372] (1:1000; #ab126598, AbCam, Cambridge UK), anti-angiotensinogen (1:1000; #ab89892, AbCam), anti-α-1-antitrypsin (EPR10832(B)) (1:1000; ab167414, AbCam) and anti-fibronectin [F1] (1:1000; #ab32419, AbCam). After incubation with the primary and HRP-conjugated secondary antibodies (1:10000 dilution; Bethyl, Montgomery, TX USA), membranes were incubated with Clarity Western ECL Substrate (Bio-Rad). Antibody-labeled proteins were detected by enhanced chemiluminescence using the Chemidoc system. The image of the gel acquired before protein transfer was used to document equal protein loading among samples.

### 4.6. Protein Functional Annotation

Functional annotation of the differentially abundant proteins was first done with DAVID 6.8 [46]. This method allowed us to identify the involved GO biological processes associated with our gene lists depending on the modified Fisher exact test *p*-value and FDR for each process. Strongly enriched annotation categories (*p* < 0.01) were considered.

Functional interpretation of the differentially abundant proteins was next done using STRING v. 10.5 database (Search Tool for the Retrieval of Interacting Genes/Proteins; string-db.org [47]). For this analysis, the *Homo sapiens* interactome was used. Each gene list of proteins more abundant in either non-relapsing or relapsing HL, from both explorative and validation groups, was uploaded separately. Default settings were used. For each network obtained, we recorded the counts of the functional enrichment gene sets based on GO biological processes at PPI enrichment *p*-value < 1.0e-16 and FDR <0.05.

### 4.7. Biological Process Classification

To characterize the molecular scenario differentiating non-relapsing from relapsing HL, we did an ad hoc analysis of the GO biological processes identified with STRING (PPI enrichment *p*-value < 1.0e-16 and FDR < 0.05). This analysis aimed to go beyond the findings of individual proteins associated with relapse to discover the biological processes predictive of relapse. First, we listed all the biological processes (BiologicalClasses&Subclasses_RawData; sheets 1, 2, 6, and 7). Then, working with the biological processes identified in the explorative and validation analyses with STRING, we manually sorted them into groups with similar GO term descriptions, forming 10 biological classes. Some biological processes were not included in these classes, because they were too general to classify (e.g., regulation of biological process, regulation of molecular function) (BiologicalClasses&Subclasses_RawData; sheet 11). One biological class with a far greater number of biological processes than the others was subdivided into 11 subclasses on the basis of GO descriptions.

For each biological class or subclass, we expressed the number of differentially abundant proteins as a percentage of the total number of differentially abundant proteins, for non-relapsing and relapsing HL separately (called “relative frequency”). This calculation was done separately for the explorative and validation analyses (BiologicalClasses&Subclasses_RawData, sheets 5 and 10).

## 5. Conclusions

Overall, our data depict two different molecular scenarios in the plasma of pediatric HL patients at diagnosis, predictive of their long-term responses to therapy. Our quantitative proteomic LC-MS/MS approach validated four proteins (α-1-antitrypsin, antithrombin III, fibrinogen β and γ chains) we previously found to be differentially abundant [10]. Bioinformatics analyses with DAVID and STRING, together with our ad hoc classification of biological processes, allowed us to identify biological processes differentially associated with relapse. At diagnosis, plasma of non-relapsing HL was enriched in proteins involved in Regulation (e.g., negative regulation of endopeptidase activity) and Lipid metabolism (e.g., negative regulation of plasma lipoprotein particle oxidation, cholesterol and phosphatidylcholine metabolic process), while plasma of relapsing HL had an abundance of proteins involved in immune system (e.g., complement activation, innate immune response), Cell and ECM organization, and their regulation.

For biomarker development, the use of panels of proteins is thought to improve the chances of clinical application [48]. Our results suggest that at diagnosis of pediatric HL, different biological processes predict the occurrence of relapse and that the 18 differentially abundant proteins are simultaneously involved in many of them. The clinical validity of these panels of candidate biomarkers will be tested in future analyses.

## Figures and Tables

**Table 1:** Clinicopathological characteristics of patients with pediatric Hodgkin lymphoma (all nodular sclerosis type) who had either a favorable (non-relapsing, NR) or unfavorable (relapsing, R) response to treatment in the LH2004 trial

Group	Disease Status	Patient no.	Sex ^a^	Age at Diagnosis, Years	Stage ^b^	Systemic Symptoms	LH2004 Therapeutic Group
Explorative	NR	1	M	16	4	Yes	3
		2	F	14	4	No	3
		3	F	15	2	No	1
	R	1	M	13	2	Yes	3
		2	F	15	2	No	3
		3	M	12	2	No	3
Validation	NR	1	M	16	4	Yes	3
		2	F	13	2	No	2
		3	F	15	2	No	1
	R	1	M	13	2	Yes	3
		2	F	15	2	No	3
		3	M	12	2	No	3

^a^ M: male, F: female; ^b^ according to [21].

**Table 2:** Differentially abundant proteins in plasma between patients with non-relapsing HL and relapsing pediatric HL, in the explorative groups

UniProtKB ID	Gene	Protein	Subcellular Localization	FC
**More abundant in non-relapsing HL**				
A0A0J9YXX1	IGHV5-10-1	Immunoglobulin heavy variable 5-10-1	secreted, cell membrane	0.80
P01861	IGHG4	Immunoglobulin heavy constant γ 4	secreted, cell membrane	0.78
P08603	CFH	Complement factor H	secreted	0.79
P02765	AHSG	α-2-HS-glycoprotein	secreted	0.79
P01871	IGHM	Immunoglobulin heavy constant mu	secreted, cell membrane	0.78
P01619	IGKV3-20	Immunoglobulin kappa variable 3-20	secreted, cell membrane	0.78
P02649	APOE	Apolipoprotein E	secreted	0.78
P02654	APOC1	Apolipoprotein C-I	secreted	0.77
P01031	C5	Complement C5	secreted	0.77
P27918	CFP	Properdin	secreted	0.76
P02766	TTR	Transthyretin°	secreted, lysosome	0.75
P00751	CFB	Complement factor B	secreted	0.73
P04196	HRG	Histidine-rich glycoprotein°	secreted	0.71
P02790	HPX	Hemopexin	secreted	0.68
P49959	MRE11	Double-strand break repair protein MRE11	nucleus	0.68
P19827	ITIH1	Inter-α-trypsin inhibitor heavy chain H1	secreted	0.68
P08697	SERPINF2	α-2-antiplasmin°	secreted	0.67
P00747	PLG	Plasminogen	secreted	0.67
Q03591	CFHR1	Complement factor H-related protein 1	secreted	0.66
P0C0L5	C4B	Complement C4-B	secreted	0.64
O75636	FCN3	Ficolin-3	secreted	0.63
P15169	CPN1	Carboxypeptidase N catalytic chain	extracellular space	0.61
P04004	VTN	Vitronectin*	extracellular space	0.61
P06396	GSN	Gelsolin	cytoskeleton, secreted	0.56
Q12805	EFEMP1	EGF-containing fibulin-like extracellular matrix protein 1°	extracellular space, extracellular matrix (ECM)	0.58
P0C0L4	C4A	Complement C4-A	secreted	0.59
O14791	APOL1	Apolipoprotein L1	secreted	0.58
P00734	F2	Prothrombin	extracellular space	0.58
P07358	C8B	Complement component C8 β chain	secreted	0.57
P10909	CLU	Clusterin	nucleus, microsome, endoplasmic reticulum, cytosol, mitochondrion, nucleus	0.56
Q08380	LGALS3BP	Galectin-3-binding protein	secreted, ECM	0.55
P23142	FBLN1	Fibulin-1°	ECM	0.54
Q06033	ITIH3	Inter-α-trypsin inhibitor heavy chain H3	secreted	0.52
P00736	C1R	Complement C1r subcomponent	secreted	0.51
Q15485	FCN2	Ficolin-2	secreted, ECM	0.50
P05546	SERPIND1	Heparin cofactor 2	endoplasmic reticulum, extracellular exosome	0.50
P02746	C1QB	Complement C1q subcomponent subunit B°	secreted	0.50
P02747	C1QC	Complement C1q subcomponent subunit C	secreted	0.48
P01591	JCHAIN	Immunoglobulin J chain	secreted	0.47
P02760	AMBP	Protein AMBP	secreted	0.46
Q9BXR6	CFHR5	Complement factor H-related protein 5	secreted	0.45
P07225	PROS1	Vitamin K-dependent protein S	secreted	0.44
P02652	APOA2	Apolipoprotein A-II	secreted	0.42
P01008	SERPINC1	Antithrombin III*°	extracellular space	0.39
P00748	F12	Coagulation factor XII	secreted	0.36
P20742	PZP	Pregnancy zone protein°	secreted	0.36
P02745	C1QA	Complement C1q subcomponent subunit A	secreted	0.31
P01019	AGT	Angiotensinogen°	secreted	0.28
A0A0C4DH68	IGKV2-24	Immunoglobulin kappa variable 2-24	secreted, cell membrane	0.26
P04180	LCAT	Phosphatidylcholine-sterol acyltransferase	secreted	0.26
P24593	IGFBP5	Insulin-like growth factor-binding protein 5	secreted	0.26
P22792	CPN2	Carboxypeptidase N subunit 2	secreted	0.26
P68871	HBB	Hemoglobin subunit β	cytosol, extracellular region, secreted	0.26
P0DP03	IGHV3-30-5	Immunoglobulin heavy variable 3-30-5	secreted, cell membrane	0.26
P08709	F7	Coagulation factor VII	secreted	0.23
P01009	SERPINA1	α-1-antitrypsin*°	secreted, endoplasmic reticulum	0.23
P19823	ITIH2	Inter-α-trypsin inhibitor heavy chain H2	secreted	0.10
Q92496	CFHR4	Complement factor H-related protein 4	secreted	0.09
P48740	MASP1	Mannan-binding lectin serine protease 1	secreted	0.08
P27169	PON1	Serum paraoxonase/arylesterase 1°	extracellular space	0.08
**More abundant in relapsing HL**				
P02751	FN1	Fibronectin°	ECM	19.61
P06702	S100A9	Protein S100-A9	cytoskeleton, extracellular region, cytoskeleton, secreted, cell membrane	15.33
P35908	KRT2	Keratin, type II cytoskeletal 2 epidermal	cytoskeleton, cytosol, endoplasmic reticulum, nucleus, cell membrane	9.45
P0DJI8	SAA1	Serum amyloid A-1 protein	secreted	5.37
Q15848	ADIPO	Adiponectin	secreted	4.73
P36955	SERPINF1	Pigment epithelium-derived factor	secreted	3.51
Q9H5I5	PIEZO2	Piezo-type mechanosensitive ion channel component 2	membrane	3.10
Q9Y490	TLN1	Talin-1°	cytoskeleton, cell membrane, cell surface	3.10
P0DJI9	SAA2	Serum amyloid A-2 protein	secreted	3.06
P09871	C1S	Complement C1s subcomponent°	extracellular space	3.05
P04264	KRT1	Keratin, type II cytoskeletal 1	cell membrane	3.02
P02753	RBP4	Retinol-binding protein 4	secreted	2.77
Q86YZ3	HRNR	Hornerin	cytoplasmic granules	2.62
P02671	FGA	Fibrinogen α chain*	secreted	2.27
P02741	CRP	C-reactive protein	secreted	2.19
P02656	APOC3	Apolipoprotein C-III	secreted	2.12
P02675	FGB	Fibrinogen β chain*°	secreted	2.06
P01700	IGLV1-47	Immunoglobulin lambda variable 1-47	secreted, membrane	2.03
P05160	F13B	Coagulation factor XIII B chain	secreted	2.03
P35527	KRT9	Keratin, type I cytoskeletal 9	cytosol, extracellular exosome, nucleus, membrane	2.02
P00450	CP	Ceruloplasmin*	secreted	2.01
P05156	CFI	Complement factor I	secreted	1.95
P10643	C7	Complement component C7	secreted	1.95
P02679	FGG	Fibrinogen γ chain*°	secreted	1.94
P07360	C8G	Complement component C8 γ chain	secreted	1.86
P02748	C9	Complement component C9	secreted	1.80
P07996	THBS1	Thrombospondin-1°	endoplasmic reticulum secreted, ECM, cell surface	1.73
P63261	ACTG1	Actin, cytoplasmic 2	cytoskeleton	1.57
IGLC2_HUMAN	IGLC2	Immunoglobulin lambda constant 2	secreted, cell membrane	1.37
P18428	LBP	Lipopolysaccharide-binding protein	secreted, cytoplasmic granule membrane	1.36
P04217	A1BG	α-1B-glycoprotein°	secreted	1.35
LV39_HUMAN	IGLV3-9	Immunoglobulin lambda variable 3-9	secreted, cell membrane	1.34

* Proteins previously found to be differentially abundant by difference gel electrophoresis [10]; **°** Proteins also found to be differentially abundant in the validation groups (Table S1). FC, fold change (log_2_ ratio in spectral counts between relapsing and non-relapsing HL).

**Table 3:** Biological processes (DAVID) and biological classes (this study) in which the 18 validated differentially abundant proteins participate.

UniProtKB ID	Protein Name (Gene Symbol) ^(a)^	Biological Processes (DAVID) (*p* < 0.01) ^(b)^	Biological Classes	Regulatory Subclasses
**More abundant in non-relapsing HL (*n* = 11)**				
P01019	Angiotensinogen (AGT)	negative regulation of endopeptidase activity, regulation of blood vessel size by renin-angiotensin	transport and homeostasis, regulation, vascularization, response, cell and ECM organization, lipid metabolism, protein metabolism	immune system, transport and homeostasis, vascularization, response, cell and ECM organization, cell death, lipid metabolism, protein metabolism, signaling
P02746	Complement C1q subcomponent subunit B (C1QB)	complement activation, proteolysis, complement activation (classical pathway), innate immune response	immune system, regulation, transport and homeostasis, response, protein metabolism	immune system, response, protein metabolism
Q12805	EGF-containing fibulin-like extracellular matrix protein 1 (EFEMP1)	NA	regulation, transport and homeostasis, response, protein metabolism	response, signaling
P23142	Fibulin-1 (FBLN1)	negative regulation of cell adhesion	regulation, transport and homeostasis, coagulation, response, cell and ECM organization, protein metabolism	immune system, transport and homeostasis, response, cell and ECM organization, protein metabolism, signaling
P04196	Histidine-rich glycoprotein (HRG)	negative regulation of endopeptidase activity, platelet degranulation, negative regulation of fibrinolysis, negative regulation of cell adhesion, fibrinolysis	immune system, regulation, transport and homeostasis, coagulation, fibrinolysis, response, cell and ECM organization	immune system, transport and homeostasis, coagulation, fibrinolysis, response, cell and ECM organization, cell death, protein metabolism, signaling
P27169	Serum paraoxonase/ arylesterase 1 (PON1)	negative regulation of plasma lipoprotein particle oxidation, cholesterol metabolic process, phosphatidylcholine metabolic process,	regulation, response, lipid metabolism	transport and homeostasis
P20742	Pregnancy zone protein (PZP)	negative regulation of endopeptidase activity	regulation	protein metabolism
P01009	α-1-antitrypsin* (SERPINA1)	acute-phase response, ER to Golgi vesicle-mediated transport, platelet degranulation, blood coagulation, negative regulation of endopeptidase activity	immune system, regulation, transport and homeostasis, coagulation, response, protein metabolism	transport and homeostasis, protein metabolism
P01008	Antithrombin III* (SERPINC1)	negative regulation of endopeptidase activity, blood coagulation	immune system, regulation, transport and homeostasis, coagulation, response, protein metabolism	coagulation, response, protein metabolism
P08697	α-2-antiplasmin (SERPINF2)	acute-phase response, negative regulation of endopeptidase activity, platelet degranulation, fibrinolysis, regulation of blood vessel size by renin-angiotensin	immune system, regulation, transport and homeostasis, coagulation, fibrinolysis, response, cell and ECM organization, protein metabolism	immune system, transport and homeostasis, coagulation, fibrinolysis, vascularization, response, cell and ECM organization, protein metabolism, signaling
P02766	Transthyretin (TTR)	retinoid metabolic process, cellular protein metabolic process,	immune system, regulation, transport and homeostasis, response, cell and ECM organization, protein metabolism	transport and homeostasis, response, signaling
**More abundant in relapsing HL (*n* = 7)**				
P04217	α-1B-glycoprotein (A1BG)	platelet degranulation	immune system, transport and homeostasis, coagulation, response	none
P09871	Complement C1s subcomponent (C1S)	proteolysis, complement activation, complement activation (classical pathway), innate immune response	immune system, regulation, response, protein metabolism	immune system, response, protein metabolism
P02675	Fibrinogen β chain* (FGB)	platelet degranulation, innate immune response, response to calcium ion, fibrinolysis, blood coagulation, fibrin clot formation, platelet aggregation, positive regulation of peptide hormone secretion, plasminogen activation, positive regulation of heterotypic cell-cell adhesion, protein polymerization, cellular protein complex assembly, ECM organization, positive regulation of exocytosis, negative regulation of endothelial cell apoptotic process, platelet activation, positive regulation of vasoconstriction, positive regulation of substrate adhesion-dependent cell spreading, negative regulation of extrinsic apoptotic signaling pathway via death domain receptors, induction of bacterial agglutination	immune system, regulation, transport and homeostasis, coagulation, fibrinolysis, response, cell and ECM organization, protein metabolism	immune system, transport and homeostasis, coagulation, vascularization, response, cell and ECM organization, cell death, protein metabolism, signaling
P02679	Fibrinogen γ chain* (FGG)	platelet degranulation, innate immune response, response to calcium ion, fibrinolysis, blood coagulation, fibrin clot formation, platelet aggregation, positive regulation of peptide hormone secretion, plasminogen activation, positive regulation of heterotypic cell-cell adhesion, protein polymerization, cellular protein complex assembly, ECM organization, positive regulation of exocytosis, negative regulation of endothelial cell apoptotic process, platelet activation, positive regulation of vasoconstriction, positive regulation of substrate adhesion-dependent cell spreading, negative regulation of extrinsic apoptotic signaling pathway via death domain receptors, induction of bacterial agglutination	immune system, regulation, transport and homeostasis, coagulation, fibrinolysis, response, cell and ECM organization, protein metabolism	immune system, transport and homeostasis, coagulation, vascularization, response, cell and ECM organization, cell death, signaling, protein metabolism
P02751	Fibronectin (FN1)	platelet degranulation, acute-phase response, ECM organization	immune system, regulation, transport and homeostasis, coagulation, response, cell and ECM organization	immune system, transport and homeostasis, response, cell and ECM organization, cell death, lipid metabolism, signaling
P07996	Thrombospondin-1 (THBS1)	ECM organization, response to calcium ion, immune response, platelet degranulation, response to calcium ion, inflammatory response	immune system, regulation, transport and homeostasis, coagulation, response, cell and ECM organization, protein metabolism	immune system, transport and homeostasis, coagulation, vascularization, response, cell and ECM organization, cell death, protein metabolism, signaling
Q9Y490	Talin-1 (TLN1)	platelet degranulation, platelet aggregation	regulation, transport and homeostasis, coagulation, response, cell and ECM organization, protein metabolism	cell and ECM organization

* Proteins previously found to be differentially abundant by difference gel electrophoresis (DIGE) [10]. NA, biological annotation not available.

## Supplementary Materials

Supplementary materials can be found at https://www.mdpi.com/1422-0067/21/6/2185/s1. Table S1. Differentially abundant plasma proteins between non-relapsing and relapsing pediatric HL samples, in the validation groups. Table S2. Biological processes in which the differentially abundant proteins participate, according to DAVID (*p* < 0.01), in the explorative and validation analyses. Table S3. Biological classes and regulatory subclasses created by sorting the GO biological processes identified as involving differentially abundant proteins into groups with similar descriptions. Figure S1. Relative frequencies of the differentially abundant proteins in 10 biological classes (a) and 11 regulatory subclasses (b) in the validation analysis of pediatric HL patients. Gene symbols above bars marked with an asterisk refer to proteins involved in those processes that were also identified in the validation analysis.

## Abbreviations

2DE	two-dimensional electrophoresis
DIGE	difference gel electrophoresis
ECM	extracellular matrix
HL	Hodgkin lymphoma
LC-MS/MS	liquid chromatography mass spectrometry
