# Supplementary material for: Proteomic Profiles and Biological Processes of Relapsed vs. Non-Relapsed Pediatric Hodgkin Lymphoma
Source: Int J Mol Sci. 2020 Mar 22;21(6):2185. doi: 10.3390/ijms21062185 (PMC7139997; doi:10.3390/ijms21062185)
Supplement: Supplementary file 1 [file ijms-21-02185-s001.pdf]

Supplementary

Table S1. Differentially abundant plasma proteins between non-relapsing and relapsing pediatric HL samples, in the validation groups

UniProtKB ID	Gene	Protein	Subcellular localization	FC
**More abundant in non-relapsing HL**				
P06727	APOA4	Apolipoprotein A-IV*	secreted	0.78
P18428	LBP	Lipopolysaccharide-binding protein	secreted	0.77
P02743	SAMP	Serum amyloid P-component	secreted	0.77
O00391	QSOX1	Sulfhydryl oxidase 1	secreted, Golgi apparatus	0.75
P63261	ACTG	Actin, cytoplasmic 2	cytoskeleton	0.75
Q9BXR6	FHR5	Complement factor H-related protein 5	secreted	0.73
P22352	GPX3	Glutathione peroxidase 3	secreted	0.73
P80108	PHLD	Phosphatidylinositol-glycan-specific phospholipase D	secreted	0.71
P02747	C1QC	Complement C1q subcomponent subunit C°	secreted	0.70
P03952	KLKB1	Plasma kallikrein	secreted	0.66
Q09666	AHNK	Neuroblast differentiation-associated protein	nucleus	0.65
P03951	FA11	Coagulation factor XI	secreted	0.65
Q15582	BGH3	Transforming growth factor-β-induced protein ig-h3	secreted, extracellular matrix	0.65
Q14520	HABP2	Hyaluronan-binding protein 2	secreted	0.65
P23142	FBLN1	Fibulin-1°	ECM	0.65
P02746	C1QB	Complement C1q subcomponent subunit B°	secreted	0.62
Q15848	ADIPO	Adiponectin	secreted	0.62
P02766	TTR	Transthyretin°	secreted, cytoplasm	0.62
Q12805	EFEMP1	EGF-containing fibulin-like extracellular matrix protein 1°	ECM	0.61
P27169	PON1	Serum paraoxonase/arylesterase 1°	extracellular region, secreted	0.60
P08697	SERPINF2	α-2-antiplasmin°	secreted	0.54
P07360	CO8G	Complement component C8 γ chain	secreted	0.49
P00748	FA12	Coagulation factor XII	secreted	0.48
P12259	FA5	Coagulation factor V	secreted	0.46
Q06033	ITIH3	Inter-α-trypsin inhibitor heavy chain H3	secreted	0.44
Q15113	PCOC1	Procollagen C-endopeptidase enhancer 1	extracellular region, secreted	0.43
P0DP03	HV335	Immunoglobulin heavy variable 3-30-5	extracellular region, secreted, plasma membrane	0.43
P04196	HRG	Histidine-rich glycoprotein°	secreted	0.42
P33151	CADH5	Cadherin-5	plasma membrane, cell junction	0.41
P48740	MASP1	Mannan-binding lectin serine protease	secreted	0.40
Q02985	FHR3	Complement factor H-related protein 3	secreted	0.36
Q96PD5	PGRP2	N-acetylmuramoyl-L-alanine amidase	secreted, membrane	0.32
P01008	SERPINC1	Antithrombin-III*°	secreted	0.28
P01019	AGT	Angiotensinogen°	secreted	0.21
P02753	RET4	Retinol-binding protein 4	secreted	0.21
Q08830	FGL1	Fibrinogen-like protein 1	secreted	0.17
P01009	SERPINA1	α-1-antitrypsin*°	secreted	0.16
P20742	PZP	Pregnancy zone protein°	secreted	0.07
**More abundant in relapsing HL**				
P01861	IGHG4	Immunoglobulin heavy constant γ 4	secreted	7.71
Q9Y490	TLN1	Talin-1°	cytoskeleton, plasma membrane, cell surface, focal adhesion	5.27
P02790	HEMO	Hemopexin	secreted	4.71
P00739	HPTR	Haptoglobin-related protein	secreted	3.71
P00751	CFAB	Complement factor B	secreted	3.65

P05155	IC1	Plasma protease C1 inhibitor	secreted	3.27
P49747	COMP	Cartilage oligomeric matrix protein	ECM	2.70
P01023	A2MG	α-2-macroglobulin	secreted	2.34
P02787	TRFE	Serotransferrin	secreted	2.25
A0A0B4J1X5	HV374	Immunoglobulin heavy variable 3-74	cell membrane, secreted	1.91
P02751	FN1	Fibronectin°	ECM	1.82
P02652	APOA2	Apolipoprotein A-II	secreted	1.80
P01042	KNG1	Kininogen-1	extracellular space	1.73
P22792	CPN2	Carboxypeptidase N subunit 2	secreted	1.73
P01011	AACT	α-1-antichymotrypsin	secreted	1.73
P07996	THBS1	Thrombospondin-1°	endoplasmic reticulum secreted, extra-cellular matrix, cell surface	1.70
P00736	C1R	Complement C1r subcomponent	secreted	1.70
P0DOX8	IGL1	Immunoglobulin lambda-1 light chain	cell membrane, secreted	1.70
P04114	APOB	Apolipoprotein B-100	secreted, cytoplasm	1.63
P01859	IGHG2	Immunoglobulin heavy constant γ 2	cell membrane, secreted	1.60
P00738	HPT	Haptoglobin	secreted	1.57
P13671	CO6	Complement component C6	secreted	1.55
P09871	C1S	Complement C1s subcomponent°	secreted	1.52
P01876	IGHA1	Immunoglobulin heavy constant α 1	cell membrane, secreted	1.50
P15169	CBPN	Carboxypeptidase N catalytic chain	extracellular space	1.46
P04217	A1BG	α-1B-glycoprotein°	secreted	1.39
P02675	FGB	Fibrinogen β chain*°	secreted	1.39
Q15485	FCN2	Ficolin-2	secreted	1.38
P01857	IGHG1	Immunoglobulin heavy constant γ 1	cell membrane, secreted	1.36
P19827	ITIH1	Inter-α-trypsin inhibitor heavy chain H1	secreted	1.36
P07357	CO8A	Complement component C8 α chain	secreted	1.33
P02679	FGG	Fibrinogen γ chain*°	secreted	1.27
Q9UK55	ZPI	Protein Z-dependent protease inhibitor	secreted	1.25
P01619	KV320	Immunoglobulin kappa variable 3-20	cell membrane, secreted	1.24

* Proteins previously found to be differentially abundant by difference gel electrophoresis [10]; ° Proteins also found to be differentially abundant in the exploratory groups (Table 2). FC, fold change (log_2 ratio in spectral counts between relapsing and non-relapsing HL)

Table S2. Biological processes in which the differentially abundant proteins participate, according to DAVID (*P*<0.01), in the explorative and validation analyses

Process	Proteins, n (%)	*P*-value	UniProtKB ID
**More abundant in non-relapsing HL - Explorative group**			
negative regulation of endopeptidase activity*	14 (29.8)	5.98E-18	P04196*, P01031, P36955, P01042, P05155, P01019*, P01008*, P01009*, P04004, Q9UK55, P19827, P19823, P0C0L5, P07225
platelet degranulation*	10 (21.3)	7.86E-12	P04196*, O00391, P01042, P05155, P01009*, P00488, Q08380, P12259, P00747, P07225
blood coagulation*	10 (21.3)	1.45E-09	P04070, P01008*, P01009*, Q9UK55, P00488, P05160, P12259, P00747, P00734, P07225
fibrinolysis*	6 (12.8)	2.43E-09	P04196*, P05155, P03952, P00747, P00734, P07225
complement activation	7 (14.9)	1.29E-07	O75636, P01031, P48740, P02746, P02745, P0C0L5, P00736
complement activation, classical pathway	7 (14.9)	2.79E-07	P01031, P01871, P05155, P02746, P02745, P0C0L5, P00736
proteolysis*	11 (23.4)	7.79E-07	O75636, P48740, P04070, P02746, P02745, P03952, P12259, P00747, P00734, P0C0L5, P00736
negative regulation of blood coagulation	4 (8.5)	4.16E-06	P01042, P04070, P04004*, P07225
receptor-mediated endocytosis	7 (14.9)	1.10E-05	P48740, O14791, O43866, P00738, P04004*, Q08380, P02790
blood coagulation, intrinsic pathway*	4 (8.5)	1.53E-05	P01042, P05155, P03952, P00734
lipid transport	5 (10.6)	5.47E-05	O14791, P02656, P02652, Q92496, P05090
regulation of complement activation	4 (8.5)	7.42E-05	P01031, P04004*, P0C0L5, P07225
lipoprotein metabolic process	4 (8.5)	1.52E-04	O14791, P02656, P02652, P04180
innate immune response*	8 (17.0)	1.55E-04	O14791, P01871, P05155, P02746, P03950, P02745, P0C0L5, P00736
negative regulation of fibrinolysis	3 (6.4)	3.26E-04	P04196*, P00747, P00734
peptidyl-glutamic acid carboxylation	3 (6.4)	3.97E-04	P04070, P00734, P07225
high-density lipoprotein particle remodeling	3 (6.4)	7.54E-04	P02656, P02652, P04180
cholesterol metabolic process	4 (8.5)	8.51E-04	O14791, P27169*, P02652, P04180
ER to Golgi vesicle-mediated transport	5 (10.6)	9.49E-04	P04070, P01009*, P12259, P00734, P07225
reverse cholesterol transport	3 (6.4)	0.001	P02656, P02652, P04180
signal peptide processing	3 (6.4)	0.002	P04070, P00734, P07225
cellular protein metabolic process*	4 (8.5)	0.004	P06396, P02766*, P00747, P00734
negative regulation of cell adhesion	3 (6.4)	0.005	P04196*, P01042, P23142*
acute-phase response*	3 (6.4)	0.005	P01009*, P00738, P00734
negative regulation of cholesterol import	2 (4.3)	0.005	P02656, P02652
negative regulation of very-low-density lipoprotein particle remodeling	2 (4.3)	0.008	P02656, P02652
**More abundant in non-relapsing HL - Validation group**			
negative regulation of endopeptidase activity*	7 (1.8)	1.41E-07	P04196*, P20742*, Q06033, P01019*, P01008*, P01009*, P08697
platelet degranulation*	6 (1.6)	1.90E-06	P04196*, O00391, Q06033, P01009*, P08697, P12259
fibrinolysis*	4 (1.1)	9.84E-06	P04196*, P08697, P03952, P00748
positive regulation of fibrinolysis*	3 (7.9)	2.38E-05	P03951, P03952, P00748

proteolysis*	8 (2.11)	5.32E-05	P48740, Q14520, P02747, Q15113, P02746, P03951, P03952, P12259
acute-phase response*	4 (1.1)	6.59E-05	P18428, P01009*, P08697, P02743
phosphatidylcholine metabolic process	3 (7.9)	1.77E-04	P27169*, P06727, P80108
blood coagulation, intrinsic pathway*	3 (7.9)	5.97E-04	P03951, P03952, P00748
innate immune response*	6 (15.8)	0.002	P18428, P02747, P02746, Q96PD5, P02743, P00748
cellular protein metabolic process*	4 (10.5)	0.02	P02766*, P06727, P02743, Q15582
negative regulation of cell adhesion*	3 (7.9)	0.003	P04196*, P23142*, Q15582
Factor XII activation	2 (5.3)	0.004	P03952, P00748
response to lipid hydroperoxide	2 (5.3)	0.004	P22352, P06727
blood coagulation*	4 (10.5)	0.006	P01008*, P01009*, P03951, P12259
negative regulation of plasma lipoprotein particle oxidation	2 (5.3)	0.006	P27169*, P06727
regulation of blood vessel size by renin-angiotensin	2 (5.3)	0.006	P01019*, P08697
retinoid metabolic process	3 (7.9)	0.007	P02753*, P02766*, P06727
**More abundant in relapsing HL - Explorative group**			
platelet degranulation*	10 (27.8)	3.97E-13	P02671, P02751*, P02679*, Q06033, P02675, Q8WZ42, P04217, P07996*, P01023, P01011
complement activation, classical pathway*	7 (19.4)	4.24E-08	P01861, P07357, P05156, P02748, P09871, P13671, P10643
innate immune response*	10 27.8	1.29E-07	P02671, P06702, P01861, P0DJI8, P05156, P02675*, P59666, P09871, P13671, Q96PD5
regulation of complement activation*	5 (13.9)	3.70E-07	P07357, P05156, P02748, P13671, P10643
acute-phase response*	5 (13.9)	1.10E-06	P02751*, P0DJI8, P0DJI9, P02741, P01011
response to calcium ion*	5 (13.9)	5.50E-06	P02671, P02679*, P02675*, Q8WZ42, P07996*
cytolysis	4 (11.1)	9.84E-06	P07357, P61626, P13671, P10643
fibrinolysis*	4 (11.1)	9.84E-06	P02671, P02679*, P02675*, P04264
blood coagulation, fibrin clot formation*	3 (8.3)	2.38E-05	P02671, P02679*, P02675*
complement activation*	5 (13.9)	2.77E-05	P01861, P07357, P09871, P13671, P10643
retina homeostasis	4 (11.1)	7.12E-05	P02788, P63261, P61626, P04264
platelet aggregation*	4 (11.1)	7.67E-05	P02671, P02679*, P02675*, P63261
positive regulation of peptide hormone secretion	3 (8.3)	1.11E-04	P02671, P02679*, P02675*
plasminogen activation	3 (8.3)	1.42E-04	P02671, P02679*, P02675*
positive regulation of heterotypic cell-cell adhesion	3 (8.3)	2.16E-04	P02671, P02679*, P02675*
complement activation, alternative pathway	3 (8.3)	3.06E-04	P07357*, P02748, P10643
protein polymerization	3 (8.3)	3.06E-04	P02671, P02679*, P02675*
cellular protein complex assembly	3 (8.3)	5.97E-04	P02671, P02679*, P02675*
ECM organization*	5 (13.9)	6.35E-04	P02671, P02751*, P02679*, P02675*, P07996*
defense response to Gram-positive bacterium	4 (11.1)	6.69E-04	P59666, P61626, Q96PD5, P02741
positive regulation of exocytosis	3 (8.3)	8.16E-04	P02671, P02679*, P02675*
negative regulation of endothelial cell apoptotic process	3 (8.3)	0.001	P02671, P02679*, P02675*
platelet activation	4 (11.1)	0.002	P02671, P02679*, P0DJI8, P02675*

cellular protein metabolic process	4 (11.1)	0.002	P02671, P02788, P0DJI8, P61626
negative regulation of endopeptidase activity*	4 (11.1)	0.002	Q06033, P01023, P01011, P08519
positive regulation of vasoconstriction	3 (8.3)	0.002	P02671, P02679*, P02675*
positive regulation of substrate adhesion-dependent cell spreading	3 (8.3)	0.002	P02671, P02679*, P02675*
negative regulation of extrinsic apoptotic signaling pathway via death domain receptors	3 (8.3)	0.002	P02671, P02679*, P02675*
positive regulation of protein secretion	3 (8.3)	0.002	P02671, P02679*, P02675*
peptidyl-cysteine S-trans-nitrosylation	2 (5.6)	0.004	P06702, P04406
inflammatory response	5 (13.9)	0.007	P06702, P61626, P07996*, P02741, P01011
induction of bacterial agglutination	2 (5.6)	0.008	P02671, P02675*
**More abundant in relapsing HL - Validation group**			
platelet degranulation*	11 (33.3)	1.90E-15	P02751*, P02679*, P02787, P01042, P05155, Q9Y490*, P02675*, P04217, P07996*, P01023, P01011
complement activation*	10 (30.3)	3.23E-14	P01619, P01861, P00751, P07357, P01857, P01859, Q15485, P09871, P13671, P00736
complement activation, classical pathway*	10 (30.3)	1.07E-13	P01619, P01861, P07357, P05155, P01857, P01876, P01859, P09871, P13671, P00736
innate immune response*	9 (27.3)	8.15E-07	P01861, P05155, P01857, P01876, P02675*, P01859, P09871, P13671, P00736
negative regulation of endopeptidase activity*	6 (18.2)	2.61E-06	P01042, P05155, P19827, Q9UK55, P01023, P01011
positive regulation of B cell activation	4 (12.1)	1.44E-05	P01861, P01857, P01876, P01859
phagocytosis, recognition	4 (12.1)	1.81E-05	P01861 P01857, P01876, P01859
receptor-mediated endocytosis	6 (18.2)	2.12E-05	P01619, P04114, P01876, P00739, P00738, P02790
proteolysis	8 (24.2)	2.81E-05	P01619, P01861, P00751, P01857, P01859, Q15485, P09871, P00736
phagocytosis, engulfment	4 (12.1)	3.58E-05	P01861, P01857, P01876, P01859
B cell receptor signaling pathway	4 (12.1)	1.33E-04	P01861 P01857, P01876, P01859
ECM organization*	5 (15.2)	4.43E-04	P02751*, P02679*, P49747, P02675*, P07996*
blood coagulation, intrinsic pathway	3 (9.1)	4.95E-04	P01042, P05155, P01023
fibrinolysis*	3 (9.1)	6.78E-04	P02679*, P05155, P02675*
regulation of complement activation*	3 (9.1)	0.001389	P00751, P07357, P13671
Fc- γ receptor signaling pathway involved in phagocytosis	4 (12.1)	0.002	P01619, P01861, P01857, P01859
acute-phase response*	3 (9.1)	0.002	P02751*, P00738, P01011
defense response to bacterium	4 (12.1)	0.002	P01861, P01857, P01859, P00738
platelet aggregation*	3 (9.1)	0.003	P02679*, Q9Y490*, P02675*
negative regulation of complement activation, lectin pathway	2 (6.1)	0.004	P05155, P01023
response to calcium ion*	3 (9.1)	0.005	P02679*, P02675*, P07996*
immune response	5 (15.2)	0.007	P01619, P07357, P01876, P07996*, P00736

blood coagulation, fibrin clot formation*	2 (6.1)	0.007	P02679*, P02675*

* Biological processes or proteins common to the explorative and validation analyses in either non-relapsing HL or relapsing pediatric HL

Table S3. Biological classes and regulatory subclasses created by sorting the GO biological processes identified as involving differentially abundant proteins into groups with similar descriptions

Biological class (no. of processes)	Included biological processes
1. Immune system (31)	acute-phase response; activation of immune response; acute inflammatory response; cute-phase response; adaptive immune response; antimicrobial humoral response; cell activation; cell chemotaxis; complement activation; complement activation, alternative pathway; complement activation, classical pathway; complement activation, lectin pathway; cytolysis; humoral immune response; immune effector process; immune response-activating signal transduction; immune system process; inflammatory response; innate immune response; leukocyte chemotaxis; leukocyte mediated immunity; leukocyte migration; leukocyte migration involved in inflammatory response; myeloid cell activation involved in immune response; myeloid leukocyte activation; neutrophil degranulation; neutrophil mediated immunity; opsonization; phagocytosis; positive chemotaxis; recognition of apoptotic cell
2. Regulation (167)	
2.1. Immune system (35)	negative regulation of chemotaxis; negative regulation of complement activation, lectin pathway; negative regulation of cytokine production; negative regulation of cytokine secretion; negative regulation of immune effector process; negative regulation of immune response; negative regulation of immune system process; negative regulation of inflammatory response; negative regulation of macrophage differentiation; neutrophil chemotaxis; positive regulation of chemokine production; positive regulation of cytokine biosynthetic process; positive regulation of cytokine production; positive regulation of cytolysis; positive regulation of humoral immune response; positive regulation of immune response; positive regulation of immune system process; positive regulation of inflammatory response; positive regulation of innate immune response; positive regulation of interleukin-8 production; positive regulation of leukocyte chemotaxis; positive regulation of macrophage activation; regulation of acute inflammatory response; regulation of complement activation; regulation of cytokine production; regulation of cytokine secretion; regulation of granulocyte chemotaxis; regulation of humoral immune response; regulation of immune effector process; regulation of immune response; regulation of immune system process; regulation of inflammatory response; regulation of innate immune response; regulation of interleukin-8 production; regulation of macrophage derived foam cell differentiation
2.2. Transport and homeostasis (37)	blood coagulation, intrinsic pathway; endoplasmic reticulum to Golgi vesicle-mediated transport; negative regulation of endocytosis; negative regulation of lipid localization; negative regulation of lipid transport; negative regulation of transport; positive regulation of anion transport; positive regulation of cholesterol efflux; positive regulation of cytolysis; positive regulation of endocytosis; positive regulation of exocytosis; positive regulation of glucose import; positive regulation of insulin secretion; positive regulation of peptide hormone secretion; positive regulation of peptide secretion; positive regulation of phagocytosis; positive regulation of protein secretion; positive regulation of receptor-mediated endocytosis; positive regulation of transport; regulated exocytosis; regulation of body fluid levels; regulation of cholesterol transport; regulation of cholesterol transport; regulation of endocytosis; regulation of hormone secretion; regulation of lipid localization; regulation of lipid transport; regulation of peptide secretion; regulation of peptide transport; regulation of protein secretion; regulation of protein transport; regulation of receptor-mediated endocytosis; regulation of secretion; regulation of secretion by

	cell; regulation of transport; regulation of vesicle-mediated transport; response to calcium ion
2.3. Coagulation (8)	negative regulation of blood coagulation; negative regulation of platelet activation; negative regulation of wound healing; positive regulation of blood coagulation; positive regulation of wound healing; regulation of blood coagulation; regulation of platelet activation; regulation of wound healing
2.4. Fibrinolysis (4)	negative regulation of fibrinolysis; positive regulation of fibrinolysis; regulation of fibrinolysis; regulation of plasminogen activation
2.5. Vascularization (8)	negative regulation of angiogenesis; positive regulation of vasoconstriction; regulation of angiogenesis; regulation of blood pressure; regulation of blood vessel diameter; regulation of blood vessel diameter by renin-angiotensin; regulation of blood vessel size; regulation of vascular associated smooth muscle cell migration
2.6. Response (13)	negative regulation of response to external stimulus; negative regulation of response to stimulus; positive regulation of defense response; positive regulation of immune response; positive regulation of response to external stimulus; positive regulation of response to stimulus; regulation of acute inflammatory response; regulation of cellular response to insulin stimulus; regulation of defense response; regulation of humoral immune response; regulation of response to external stimulus; regulation of response to stimulus; regulation of response to stress
2.7. Cell and ECM organization (21)	cell-substrate junction assembly; extracellular structure organization; negative regulation of cell adhesion; negative regulation of cell-substrate adhesion; negative regulation of cellular component organization; positive regulation of cell adhesion; positive regulation of cell junction assembly; positive regulation of cell-substrate adhesion; positive regulation of cellular component organization; positive regulation of collagen biosynthetic process; positive regulation of collagen metabolic process; positive regulation of heterotypic cell-cell adhesion; positive regulation of substrate adhesion-dependent cell spreading; positive regulation of supramolecular fiber organization; regulation of anatomical structure morphogenesis; regulation of anatomical structure size; regulation of cell adhesion; regulation of cell-cell adhesion mediated by cadherin; regulation of cell-substrate adhesion; regulation of cellular component organization; regulation of heterotypic cell-cell adhesion
2.8. Cell death (12)	negative regulation of cell death; negative regulation of endothelial cell apoptotic process; negative regulation of extrinsic apoptotic signaling pathway; negative regulation of extrinsic apoptotic signaling pathway via death domain receptors; negative regulation of tumor necrosis factor production; positive regulation of apoptotic cell clearance; positive regulation of cell death; recognition of apoptotic cell; regulation of apoptotic signaling pathway; regulation of cell death; regulation of endothelial cell apoptotic process; regulation of extrinsic apoptotic signaling pathway via death domain receptors
2.9. Lipid metabolism (13)	chylomicron remodeling; high-density lipoprotein particle remodeling; negative regulation of lipid biosynthetic process; negative regulation of lipid catabolic process; positive regulation of cholesterol esterification; positive regulation of fatty acid metabolic process; positive regulation of lipase activity; positive regulation of lipid metabolic process; regulation of fatty acid biosynthetic process; regulation of fatty acid metabolic process; regulation of lipid biosynthetic process; regulation of plasma lipoprotein particle levels; regulation of steroid metabolic process
2.10. Protein metabolism (38)	negative regulation of catalytic activity; negative regulation of cellular protein metabolic process; negative regulation of endopeptidase activity; negative regulation of hydrolase activity; negative regulation of protein activation cascade; negative regulation of protein processing; negative regulation of proteolysis; positive

	regulation of catalytic activity; positive regulation of cellular protein metabolic process; positive regulation of oxidoreductase activity; positive regulation of peptide secretion; positive regulation of protein metabolic process; positive regulation of protein phosphorylation; positive regulation of protein processing; positive regulation of proteolysis; protein metabolic process; regulation of catalytic activity; regulation of cellular protein metabolic process; regulation of endopeptidase activity; regulation of hydrolase activity; regulation of lipase activity; regulation of peptidase activity; regulation of peptide secretion; regulation of peptidyl-tyrosine phosphorylation; regulation of phosphorylation; regulation of protein activation cascade; regulation of protein binding; regulation of protein complex assembly; regulation of protein homooligomerization; regulation of protein metabolic process; regulation of protein oligomerization; regulation of protein phosphorylation; regulation of protein processing; regulation of protein secretion; regulation of protein stability; regulation of protein transport; regulation of proteolysis; regulation of tau-protein kinase activity
2.11. Signaling (16)	negative regulation of ERK1 and ERK2 cascade; negative regulation of MAPK cascade; negative regulation of tumor necrosis factor production; positive regulation of ERK1 and ERK2 cascade; positive regulation of intracellular signal transduction; positive regulation of MAPK cascade; positive regulation of receptor signaling pathway via JAK-STAT; positive regulation of signal transduction; positive regulation of signaling; positive regulation of tumor necrosis factor biosynthetic process; regulation of ERK1 and ERK2 cascade; regulation of intracellular signal transduction; regulation of signal transduction; regulation of signaling; regulation of tumor necrosis factor production; toll-like receptor signaling pathway
3. Transport and homeostasis (34)	cellular chemical homeostasis; cellular iron ion homeostasis; cellular transition metal ion homeostasis; chemical homeostasis; cholesterol efflux; cholesterol homeostasis; cholesterol transport; COPII vesicle coating; cytolysis; endocytosis; endoplasmic reticulum to Golgi vesicle-mediated transport; glucose homeostasis; heme transport; iron ion transport; lipid transport; localization; macromolecule metabolic process; negative regulation of cholesterol transport; nitrogen compound metabolic process; organic substance metabolic process; organonitrogen compound metabolic process; phagocytosis; phospholipid efflux; plasma lipoprotein particle clearance; positive regulation of cholesterol efflux; positive regulation of exocytosis; protein import; receptor-mediated endocytosis; regulated exocytosis; reverse cholesterol transport; reverse cholesterol transport; secretion; secretion by cell; transport
4. Coagulation (9)	blood circulation; blood coagulation; blood coagulation, fibrin clot formation; blood coagulation, intrinsic pathway; Factor XII activation; platelet activation; platelet aggregation; platelet degranulation; wound healing
5. Fibrinolysis (2)	fibrinolysis; plasminogen activation
6. Vascularization (5)	blood circulation; blood vessel development; blood vessel morphogenesis; circulatory system development; vasodilation
7. Response (31)	cellular response to chemical stimulus; cellular response to cytokine stimulus; cellular response to organic substance; cellular response to stimulus; defense response; defense response to bacterium; response to acid chemical; response to activity; response to bacterium; response to calcium ion; response to carbohydrate; response to chemical; response to cytokine; response to external stimulus; response to glucose; response to growth hormone; response to hormone; response to inorganic substance; response to lipid; response to lipid hydroperoxide; response to mechanical stimulus; response to metal ion; response to misfolded protein; response to nutrient level; response to organic substance; response to other organism; response to oxygen-containing compound; response to steroid hormone; response to stimulus; response to stress; response to toxic substance
8. Cell and ECM organization (12)	cell adhesion; cell junction assembly; cell-cell adhesion; cell-matrix adhesion; cell-substrate junction assembly; cellular component assembly; cellular component organization; cytoskeleton organization; ECM organization; extracellular structure organization; integrin activation; regulation of cell-substrate adhesion

9. Lipid metabolism (24)	acylglycerol catabolic process; cellular lipid metabolic process; cholesterol homeostasis; cholesterol metabolic process; chylomicron assembly; chylomicron remnant clearance; chylomicron remodeling; glycerolipid metabolic process; high-density lipoprotein particle assembly; high-density lipoprotein particle clearance; high-density lipoprotein particle remodeling; lipoprotein metabolic process; low-density lipoprotein particle remodeling; negative regulation of lipid metabolic process; phosphatidylcholine biosynthetic process; phosphatidylcholine metabolic process; plasma lipoprotein particle assembly; plasma lipoprotein particle clearance; plasma lipoprotein particle remodeling; positive regulation of cholesterol esterification; triglyceride metabolic process; triglyceride-rich lipoprotein particle remodeling; very-low-density lipoprotein particle clearance; very-low-density lipoprotein particle remodeling
10. Protein metabolism (12)	negative regulation of catalytic activity; negative regulation of lipase activity; peptide cross-linking; post-translational protein modification; protein activation cascade; protein metabolic process; protein polymerization; protein processing; protein-containing complex assembly; protein-containing complex subunit organization; proteolysis; zymogen activation

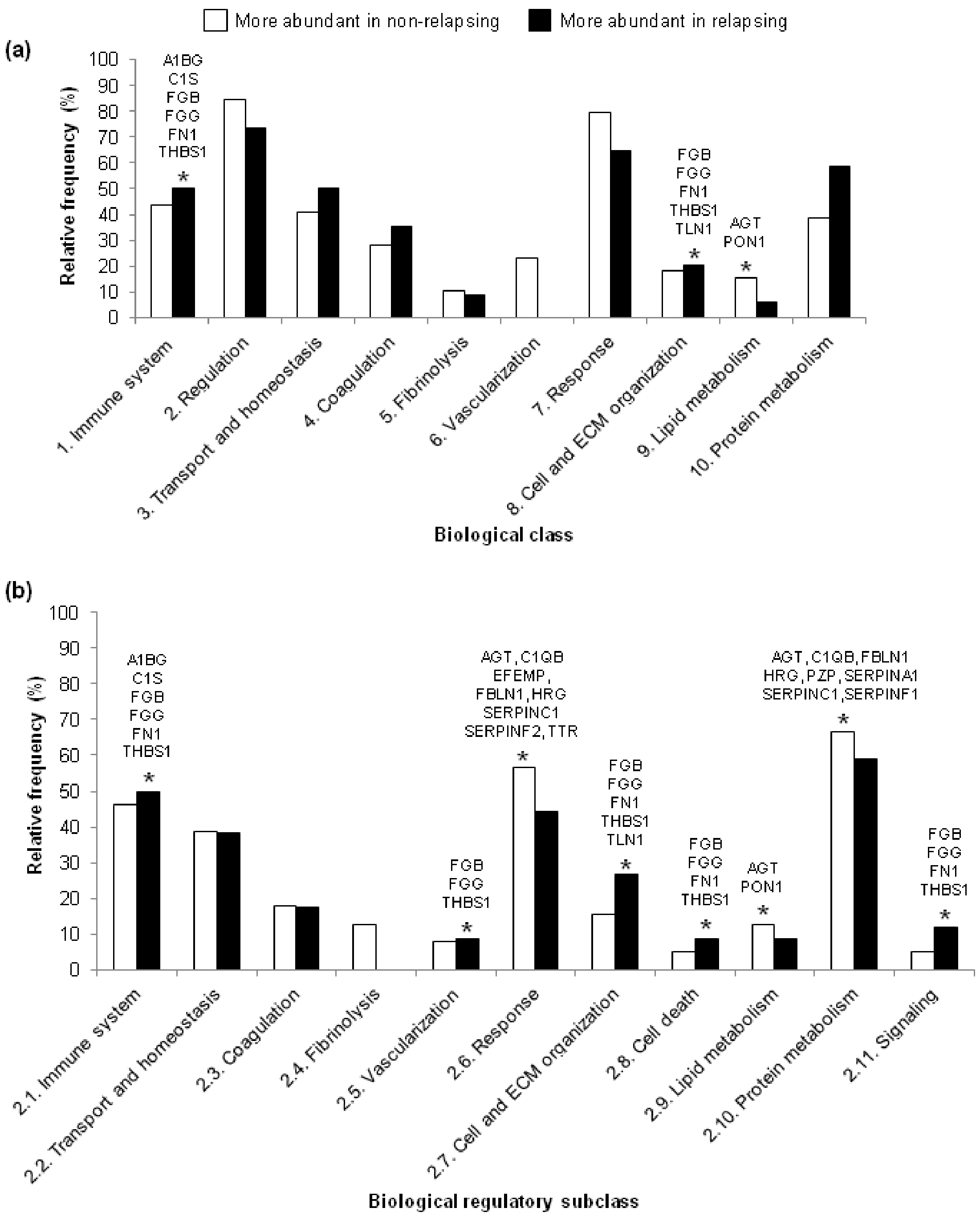

Figure S1. Relative frequencies of the differentially abundant proteins in 10 biological classes (**a**) and 11 regulatory subclasses (**b**) in the validation analysis of pediatric HL patients. Gene symbols above bars marked with an asterisk refer to proteins involved in those processes that were also identified in the validation analysis.
